# Investigating the Effects of Exam Length on Performance and Cognitive Fatigue

**DOI:** 10.1371/journal.pone.0070270

**Published:** 2013-08-12

**Authors:** Jamie L. Jensen, Dane A. Berry, Tyler A. Kummer

**Affiliations:** Department of Biology, Brigham Young University, Provo, Utah, United States of America; Universidade de Brasília, Brazil

## Abstract

This study examined the effects of exam length on student performance and cognitive fatigue in an undergraduate biology classroom. Exams tested higher order thinking skills. To test our hypothesis, we administered standard- and extended-length high-level exams to two populations of non-majors biology students. We gathered exam performance data between conditions as well as performance on the first and second half of exams within conditions. We showed that lengthier exams led to better performance on assessment items shared between conditions, possibly lending support to the spreading activation theory. It also led to greater performance on the final exam, lending support to the testing effect in creative problem solving. Lengthier exams did not result in lower performance due to fatiguing conditions, although students perceived subjective fatigue. Implications of these findings are discussed with respect to assessment practices.

## Introduction

Effective assessment is one of the most challenging aspects of teaching. According to backward design [1], assessments should test for achievement of desired learning outcomes. In addition, each outcome should be measured by multiple items to achieve reliable feedback on students' knowledge [2]. This brings up many questions: How many items are optimal to effectively assess each learning outcome? Does including more items give us a more accurate assessment instrument or is it redundant? Do students experience cognitive fatigue causing their score to suffer with longer exams?

As test length increases, two concerns arise. First, is the task causing cognitive fatigue; in other words, is it taxing students' mental functions enough that it may cause a decrease in their performance? Second, is the task causing perceptions of fatigue, or subjective fatigue [3]? More importantly, is this subjective fatigue leading to a self-regulated withdrawal from the process and consequently lower performance? In both cases, fatiguing conditions can lead to lower student performance not attributable to a lack of knowledge, and consequently less than accurate assessment outcomes. A literature review [4] revealed that most research on the subject of cognitive or mental fatigue is older, predating the 1950s [5], [6]. In addition, the findings are mixed. Martyn [7] found a decline in performance as more items and greater time were added. Davis [8] found three different effects in students on repetitive cognitive tasks: some increased performance with more time on task, while some remained constant, and others decreased their performance. However, this study was done using a set of repetitive tasks and does not directly transfer to a test of conceptual understanding.

When the SAT increased in length in 2005, high school guidance counselors complained that scores would drop due to fatiguing conditions with the increased length [9]. In fact, it is popular belief and rather intuitive that increased test length would lead to lower performance [3], [10]. However, Laitusis, Morgan, Bridgeman, Zanna, and Stone [11] found that students taking the SAT under extended-time conditions (i.e., more time-on-task, but not more items) experienced no difference in exam performance. Liu, Allspach, Feigenbaum, Oh, and Burton [12] reviewed research on the effects of additional items being added to a test and concluded that repetitive, low-stakes items are affected by fatigue; whereas, high-stakes, complex, and varied items do not seem to be affected. Ackerman and Kanfer [3] compared performance of students on the SAT exam in a shortened, standard, and lengthened condition. The conditions varied in the number of items given and consequently in the time-on-task. Interestingly, they found that increased test length (i.e., more test items) led to significantly increased test scores. Most of these studies were run on context-independent, ability-based high stakes tests, such as the SAT. Whereas, research on the effects of test length on students' ability to demonstrate conceptual understanding in a context-specific framework (i.e., biology) is absent.

The testing effect is the well-demonstrated idea that recalling information in a testing situation is more beneficial than re-reading or re-studying the information [13]. Thus, lengthier tests, despite being possibly fatiguing, may enhance the testing effect on the subsequent final exam. However, much research on the testing effect has been done in artificial laboratory environments, rather than classroom settings, and most often involves the recall of facts rather than the application of concepts in novel situations requiring higher-order thinking skills [14]–[22].

This study compares performance on a final exam requiring the application of biological concepts to novel situations after initial unit exam testing. This study also investigates whether students experience cognitive fatigue and if exams can be shortened and still effectively assess students' understanding. We predicted that, after assessing each learning outcome with at least two items, additional items would simply cause increased cognitive load leading to lower performance and less accurate assessment of students' understanding. An alternative prediction, however, is that additional items actually increase student performance and provide a more accurate assessment of student understanding.

## Methods

### Ethics Statement

Permission for human subjects use was obtained by the Brigham Young Institutional Review Board for Human Subjects and written consent was obtained from all participants.

### Course and Participants

The subject participants were undergraduate students enrolled in two 70+ sections of non-majors general biology at a large private University. The course, which met three one-hour periods per week, is part of the general education required core and covers the entire biology curriculum, from molecular and cellular biology, to genetics and biotechnology, to evolution and ecology. Both sections were taught in the same inquiry fashion using the learning cycle [23], [24], specifically, exploratory activities to introduce each unit followed by term introduction and concept application activities. Homework assignments were identical between sections. Students enrolled in the course were majority non-science majors and ranged from freshman to seniors.

### Experimental Design

A quasi-experimental nonequivalent groups design was utilized. Steps were taken to ensure as much group equivalence as possible among the two treatment groups. To do so, identical classrooms, course materials, textbooks, resources, curriculum, and expected learning outcomes were utilized. The same instructor taught both treatment conditions. Finally, pre-assessments for reasoning skills and prior biological knowledge were administered to detect any differences in groups. Exams were multiple-choice and were written to assess deep conceptual understanding and critical thinking skills. Items were written at the “Apply,” “Analyze,” and “Evaluate” levels of Bloom's Taxonomy [25], [26]. Items were classified by three independent researchers trained in Bloom's taxonomy. Generally, exams consisted of biological scenarios followed by questions asking students to analyze data, apply concepts, and evaluate solutions to each scenario. The *standard* treatment condition was administered three unit exams consisting of 50 multiple-choice items administered in the University Testing Center with no time constraints (N = 76). The *extended* treatment condition was administered three unit exams consisting of 100 multiple-choice items (N = 79). The 50-item exams contained at least two questions covering each expected learning outcome for that unit. The 100-item exams consisted of these same 50 items plus 50 additional questions consisting of more items added to existing scenarios and/or additional scenarios for each concept. A sample scenario with accompanying questions in each treatment condition is illustrated below.

#### Scenario

You and your spouse are expecting your first child. Because you and your spouse have an extensive history of genetic diseases on both sides of the family, you are concerned about the health of your unborn baby and request that an amniocentesis be performed so that you can prepare yourself for whatever lies ahead. During this procedure, a large needle is inserted through the mother's belly and into the amniotic sack in order to collect a sample of amniotic fluid containing sloughed cells from the embryo. These cells are then grown in a laboratory for about a week in order to analyze the chromosomes.

#### Items included on the standard condition only

1. The DNA from a typical human sperm cell weighs approximately 3.3 picograms (a picogram is a 10^−12^ gram). If all chromosomes weighed approximately the same, how much does a typical chromosome weigh?
  - 0.07 picograms
  - *0.14 picograms*
  - 0.28 picograms
  - 75.9 picograms
  - 151.8 picograms
2. Doctors find that in the majority of the embryo's cells, the DNA weighs over 6.7 picograms. What is a possible explanation for this?
  - The embryonic cells remained haploid, meaning that the sperm's chromosomes never fused with the egg's and were most likely kicked out with the polar body.
  - The embryonic cells contain all duplicated chromosomes; thus, there must be a problem with Meiosis II and sister chromatids are never separating.
  - *The embryonic cells contain extra DNA indicating a possible trisomy.*
  - The embryo is normal but is definitely a girl, since girls carry an extra X chromosome.
3. If I have a chromosome #3 from the embryo and I compare it to a chromosome #3 from myself, what should I expect?
  - They should be exactly the same since they are identical chromosomes.
  - *My chromosome will be slightly lighter in weight than the embryo's because mine are older and have shorter telomeres.*
  - My chromosome will be slightly heavier in weight than the embryo's chromosomes because mine are more mature and therefore have more base-pairs.
  - The embryo's chromosome should be half the weight of my chromosome since my chromosome split in half to make the haploid cell that I donated to the embryo.

#### Additional items included on the extended condition

1. In which phase of the cell cycle would doctors analyze the cell's contents in order to look for chromosomal abnormalities?
  - During G_1_ phase
  - During S phase
  - During G_2_ phase
  - *During the Mitotic phase*
  - During the Meiotic phase
2. If the doctors want to analyze the DNA of a cell in G_2_ phase, right before mitosis takes place, how much should the cell's DNA weigh?
  - 3.3 picograms
  - 6.6 picograms
  - *13.2 picograms*
  - 26.4 picograms
3. The banding patterns (the light and dark lines that you see on the chromosomes when you dye them) indicate regions of actively transcribed (light, i.e. gene sequences) and never transcribed (dark, i.e. “junk” sequences) areas of the chromosome. If I compared the banding patterns on my chromosome #3 to the banding patterns on the embryo's chromosome #3, what should I expect?
  - *They should have identical banding patterns since they are carrying the same set of genes (but not necessarily the same alleles) in the same locations.*
  - They may be slightly different in banding patterns because the embryo's chromosome #3 may be carrying different alleles in different locations than my chromosome #3.
  - They will most definitely have different banding patterns since they are not homologous chromosomes.
  - They may have identical banding patterns on parts of the chromosomes but different on other parts due to crossing-over with non-homologous chromosomes.

Both sections took a common final exam that consisted of 21 high-level items, 20 low-level items requiring simple information recall to test students' ability to recall factual information, along with the LCTSR and IMCA (described in section 2.3.1 and 2.3.2) interspersed. The content items were comprehensive and allowed us to test for common understanding between treatment conditions.

### Dependent Measures

#### Initial reasoning ability and reasoning gains

A modified version of Lawson's Classroom Test of Scientific Reasoning (LCTSR; ver. 2000) [27] consisting of 24 items was used to assess initial reasoning ability and reasoning gains. The test measures several constructs of scientific reasoning ability (e.g., conservation of mass, probabilistic reasoning, correlational reasoning, controlling variables, and hypothetico-deductive reasoning). Results of the test indicate at what stage of Piaget's epistemological hierarchy students are, from concrete reasoners, to formal reasoners, to post-formal reasoners. Scoring procedures, validity and reliability of the test are discussed in Lawson, Alkhoury, Benford, Clark, and Falconer [28]. Briefly, scores from 0–8 are level 3, or concrete operational thinkers. Scores from 9–14 are low level 4, or students transitioning from concrete to formal operations. Scores from 15–20 are high level 4, or students transitioning from formal to post-formal operations. Scores from 21–24 are level 5, or post-formal operational thinkers. The reasoning test was administered as an in-class assignment at the beginning of the course and given five points for its completion (out of a possible 895 points in the course), regardless of score. It was then administered in conjunction with the final exam at the end of the course and graded according to performance. Reasoning gains were calculated by subtracting initial scores from final scores. Gains were reported as a positive number; digression was reported as a negative number. Initial reasoning was assessed to test for group equivalence. Reasoning gains were assessed to determine if the treatment conditions affected the development of critical thinking skills.

#### Biological understanding

Student achievement of expected learning outcomes was assessed using three unit exams. Exams in both conditions were worth 200 points and were reported as raw percentages (i.e., number of items correct out of total number of items). Exam items in the extended condition were each worth two points; exam items in the standard condition were each worth four points. Unit exams comprised 50% of the students' final grades.

Student understanding was also assessed by a common final exam. The exam, informed by several standardized biology exams, consisted of 20 low-level multiple-choice items and 21 high-level multiple-choice items. Students were assigned a low-level achievement score by averaging their performance on the 20 low-level items and a high-level achievement score by averaging their performance on the 21 high-level items. Items were designed and then categorized into Bloom levels by three independent researchers trained in assessing levels of Bloom's Taxonomy. Items were discussed and modified until all raters came to an agreement on the Bloom's level. Internal consistency of test items was also assessed using Cronbach's alpha as was determined to be .66 for the 41 content questions. Because so many different constructs were being measured by this exam, reliability was difficult to determine and overall internal consistency was not expected to be high.

In addition, students were given the Introductory Molecular and Cellular Biology Assessment (IMCA) [29] at the beginning and end of the course. The IMCA is a published inventory of basic biological content including molecular biology, cellular biology, genetics, and gene expression. Validity and reliability of the IMCA has been established [29].

#### Test-taking time

Students were electronically admitted and discharged from the University Testing Center, a center that proctors exams on campus. Thus, time spent taking each exam was recorded for each student. Time spent on each unit exam was averaged for each treatment condition.

#### Student comments

Comments about the course were collected from both treatment conditions through anonymous course evaluations administered by the University. No specific prompts were given for student impressions of assessment. Comments specifically addressing the length of assessments were unsolicited and were therefore considered genuine and candid impressions.

## Results

### Initial Reasoning Ability and Reasoning Gains

An independent-samples t-test of LCTSR scores at the beginning of the semester indicated that sections were equally matched in initial reasoning ability (see Table 1) and that, on average, all students had achieved formal reasoning ability. Despite this high achievement, students still had room to improve. Both sections showed significant improvement (*p*<.01) in final LCTSR scores when compared to initial LCTSR scores, and reached an equal level of mastery (each increasing by nearly two points, see Table 1).

**Table 1:** Results of the LCTSR and IMCA given at the beginning (pre) and end (post) of the semester.

	Standard Condition (*N* = 76)	Extended Condition (*N* = 79)	*t (df)*	*p*
Pre LCTSR	18.4	19.4	1.73 (153)	NS
Post LCTSR	20.1	21.0	1.66 (153)	NS
*t (df)*	4.59 (75)	5.35 (78)		
*p*	<.01	<.01		
Pre IMCA	35.5	40.0	2.10 (153)	.038
Post IMCA	55.9	61.2	2.10 (153)	.037
*t (df)*	10.9 (75)	13.1 (78)		
*p*	<.01	<.01		

The LCTSR has 24 items and is scored from 1 to 24. The IMCA consists of 24 items and is scored as a total percentage correct.

### Biological Understanding

#### IMCA

Results of the IMCA given at the beginning of the semester showed very low prior knowledge of biology. However, an independent-samples t-test showed that students in the extended condition had slightly higher scores than students in the standard condition (see Table 1). Both conditions made significant improvement shown by post-test scores; however, neither condition demonstrated C-level achievement of the concepts on the IMCA, evidenced by scores less than 70% (see Table 1).

#### Unit exams

**Figure 1:**
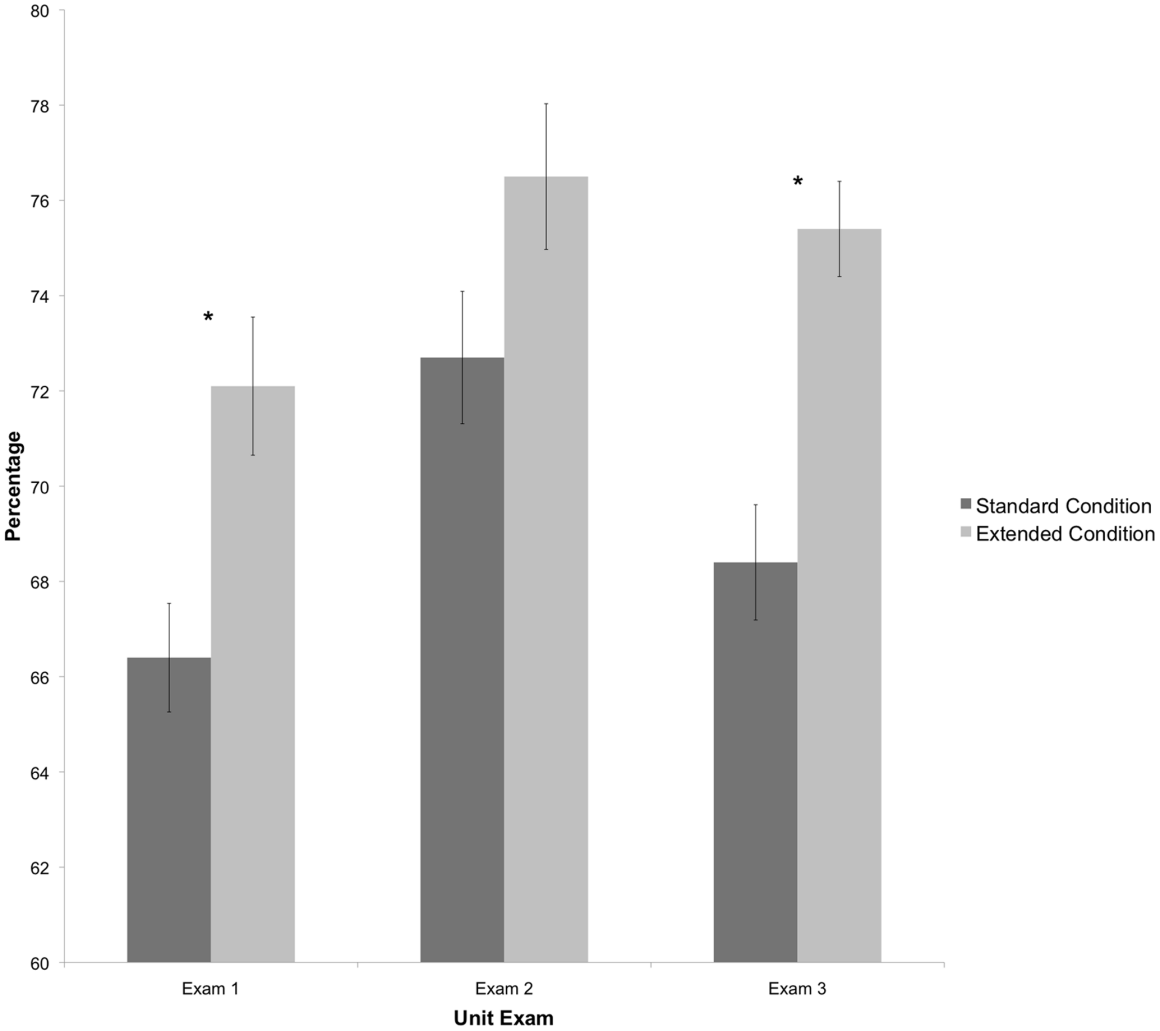
Raw percentages on each unit exam for each treatment condition. ** *p*<.01.

Raw percentages (number of items correct out of the total number of items) on unit exams in each section are shown in Figure 1. Because prior biology exposure differed between sections, as evidenced by different pre IMCA scores, an analysis of covariance, with pre IMCA scores as a covariate, was run to control for these differences. On each unit exam, students in the extended condition achieved higher percentages than students in the standard condition, although exam 2 differences were not significant (see Table 2 and Figure 1). The question then became whether the 50 extra questions included in the extended condition were possibly easier and were therefore causing score inflation. To test this, a paired-samples t-test was run comparing performance on the 50 shared questions to performance on the 50 additional questions in the extended treatment only. On exam 1, the shared questions were more difficult (*M*_shared_ = 71.4, *M*_additional_ = 74.0, *t*(78) = 2.50, *p* = .015). On exam 2, the shared questions were easier (*M*_shared_ = 78.1, *M*_additional_ = 75.0, *t*(78) = 4.35, *p*<.01). On exam 3, the shared questions were more difficult (*M*_shared_ = 74.4, *M*_additional_ = 76.0, *t*(78) = 2.39, *p* = .019).

**Table 2:** Comparison of exam performance between treatment conditions.

	Standard Condition (*N* = 76)	Extended Condition (*N* = 79)	*F(df)*	*p*
*Raw Exam Percentages*				
Exam 1	66.4	72.1	8.11(1,152)	<.01
Exam 2	72.7	76.5	1.76(1,152)	.19
Exam 3	68.4	75.4	15.07(1,152)	<.01
*Performance on 50 shared Items*				
Exam 1	66.4	71.4	5.49(1,152)	.02
Exam 2	72.7	78.1	4.25(1,152)	.04
Exam 3	68.4	74.4	8.28(1,152)	<.01

*Raw exam percentages* are the total score achieved on the entire exam. *Performance on 50 shared items* represents the total score on the entire exam for the standard treatment, and the total score on the 50 shared items included within the 100-question exam in the extended treatment. *F*-statistic and *p*-value for ANCOVA, using pre IMCA scores as a covariate, are reported.

**Figure 2:**
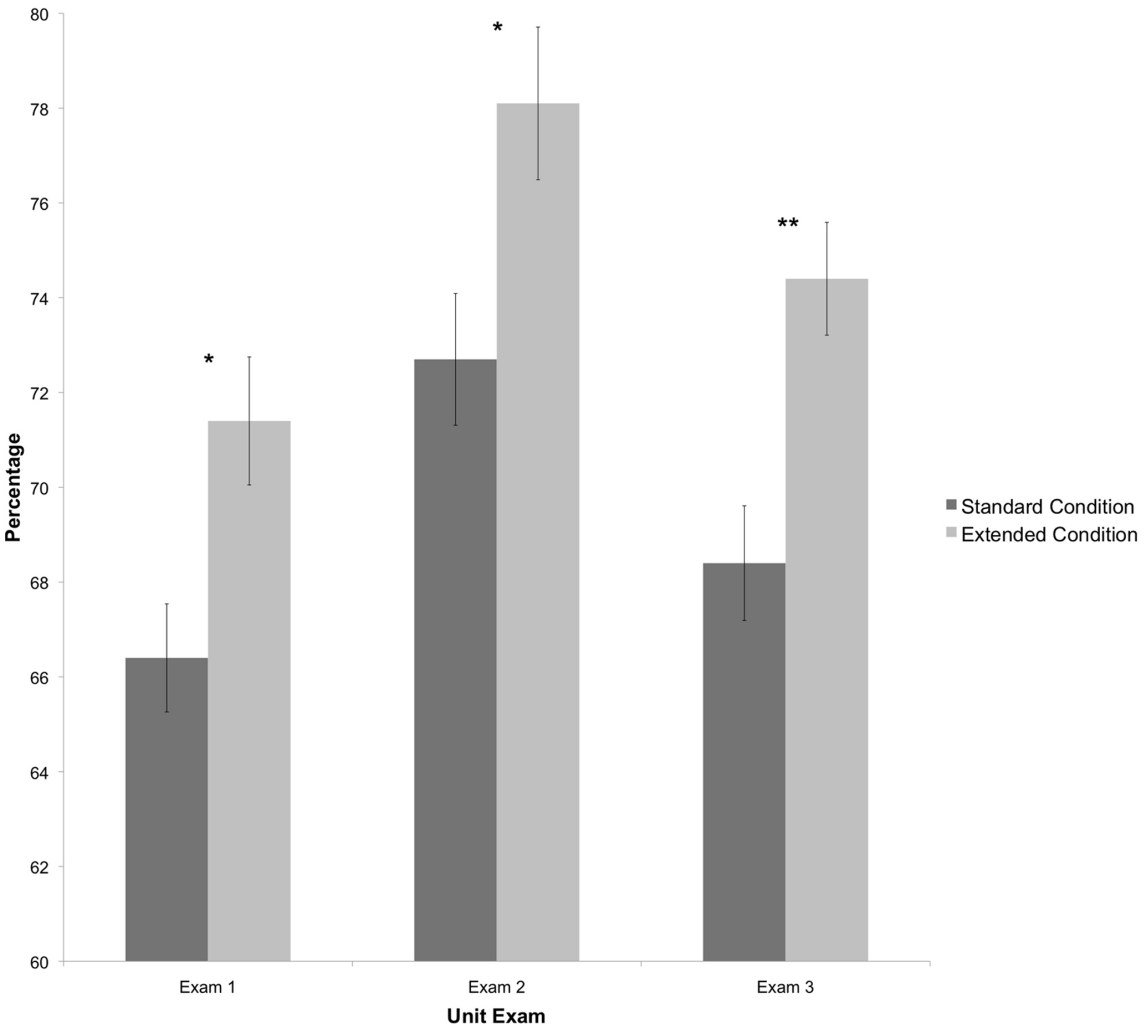
Performance on 50 shared items. These items make up the entirety of the exam for the standard condition and are interspersed throughout the extended exam. * *p*<.05, ** *p*<.01.

To test whether the 50 additional questions merely inflated the grade or actually improved performance on the 50 shared questions, an analysis of covariance was run comparing performance on the 50 shared questions between sections, using pre IMCA scores as a covariate. These questions made up the entirety of the standard condition exams and were interspersed through the test in the extended condition. On all three unit exams, students in the extended condition significantly outperformed students in the standard condition (see Table 2 and Figure 2).

#### Final exam

**Figure 3:**
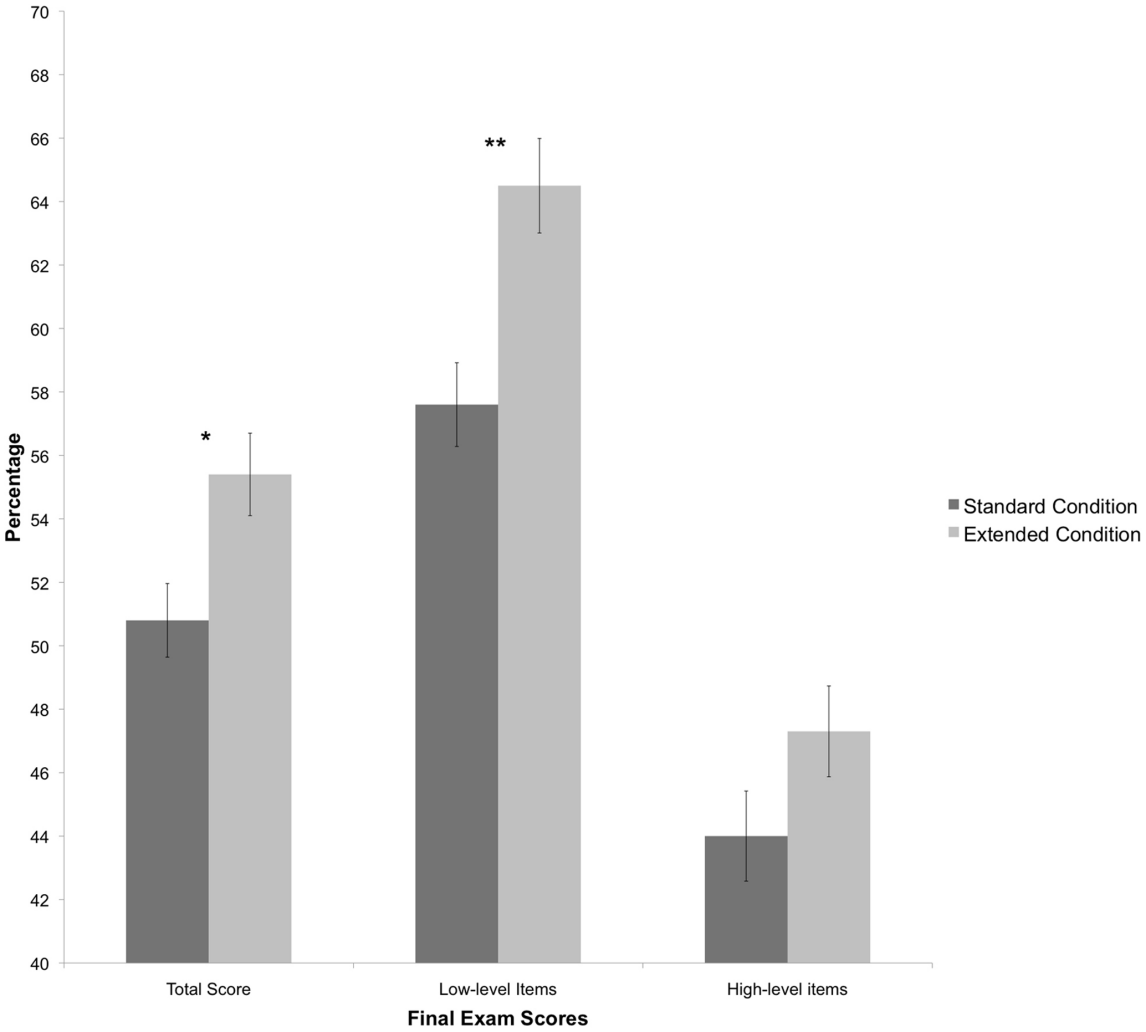
Final exam scores for each treatment condition. The total score is the average score on 41 comprehensive questions included on the final exam. Low-level items include 20 items requiring only recall of information. High-level items include 21 items requiring application, analysis, and evaluation. ** *p*<.01.

The final exam consisted of 41 comprehensive items that covered material from the entire curriculum. Of those items, 20 were low-level recall items and 21 were high-level application, analysis, and evaluation items. Students were given an average score overall and then an average score for low-level items and an average score for high-level items. An analysis of covariance, again using pre IMCA scores as a covariate, was conducted to evaluate differences in performance between treatment conditions. Students in the extended condition outperformed students in the standard condition on overall content knowledge (*M*_standard_ = 50.8%, *M*_extended_ = 55.4%, *F*(1,152) = 4.09, *p* = .05; see Figure 3). On low-level items, students in the extended condition outperformed students in the standard condition (*M*_standard_ = 58.0%, *M*_extended_ = 64.0%, *F*(1,152) = 8.83, *p*<.01; see Figure 3). On high-level items, the difference was not significant (*M*_standard_ = 44.0%, *M*_extended_ = 47.3%, *p* = NS).

### Cognitive Fatigue

To determine if students in the extended condition were experiencing cognitive fatigue during the lengthy exam, a paired-samples t-test was conducted to compare performance on the first 50 questions to performance on the second 50 questions on each unit exam. On the first and third exams, students performed equally on both halves (*p* = NS). On the second exam, students performed better on the second half than the first half (*M*_first 50_ = 74.0%, *M*_second 50_ = 79.0%, *t*(78) = 2.75, *p*<.01).

### Test-taking Time

The University Testing Center digitally records the time that each student spends taking the exam. On average, students in the standard condition spent 88 minutes in the testing center; whereas, students in the extended condition spent an average of 125 minutes in the testing center. A Pearson correlation coefficient was calculated between each unit exam within each section and the time spent taking the exam. No significant correlations were found on any exam in either section indicating that time spent in the testing center had no relationship to the score earned.

## Discussion

### Reasoning Ability

LCTSR scores indicated that groups were equally matched in initial reasoning ability, eliminating reasoning skills as a causal mechanism for improved performance by students in the extended condition. In addition, even though both groups began with formal reasoning skills, both made significant improvements in reasoning ability going up an average of two points. Exam length differences appeared to have little effect on improving reasoning ability and improvement was more likely a result of the inquiry-based instructional strategy shared by both treatments. Inquiry instruction has been shown to be effective in improving reasoning ability [30]–[36].

### Biological Understanding

IMCA scores suggested that the students in the extended condition had, on average, more prior knowledge of biological concepts entering the course. However, scores were still very low. Even with significant improvements in both classes (about 20% in both classes), neither class averaged above a 62% on the IMCA. This is likely due to the in-depth nature of this inventory, having been written for biology majors. Concepts assessed by the IMCA were not covered in detail during the course and much of the terminology would have been unfamiliar to students. In addition, the IMCA covered only the molecular, cellular, and genetics sections of the curriculum and therefore did not give a complete assessment of students' knowledge of the full curriculum. However, to control for any possibility that these difference had a causal effect, the pre IMCA scores were used as a covariate in the ANCOVA analyses.

Unit exams were designed to test the same set of learning outcomes in both sections. The difference between treatments was the number of questions assessing each outcome with the extended condition having twice as many. Students in the extended condition consistently outperformed students in the standard condition reflected by higher exam scores and consequently higher course grades. However, results indicated that the 50 shared items and the 50 additional items were not equal in difficulty. On the first and third exam, the additional items were easier, evidenced by better performance on these than the shared items by the extended section that took both sets of items. This led us to suspect that perhaps the additional items were inflating the scores of students in the extended condition by giving them easier questions to answer. To test this hypothesis, we compared performance on the 50 shared items alone. If our hypothesis had been correct, we would have expected to see equal performance on these shared items by both sections. However, students in the extended condition consistently outperformed students in the standard condition on these shared items. This suggests that students in the extended condition somehow had an advantage on these items, despite the common curriculum, classroom, and instructor between them. Because the length of the exam was the only difference between sections, we concluded that additional items provided an advantage.

The testing effect would only render an advantage in a re-test situation and should have no effect on initial performance unless students in the extended condition participated in more testing practice prior to the exam, a fact of which we have no evidence. Therefore, superior performance on the same set of items during the actual initial testing event cannot be explained by the testing effect.

This led us to two possible explanations: first, these additional items aid in cognitive recall by providing more recall cues helping students to remember more of the material they learned in class; second, additional items provided further context to the 50 shared questions increasing student understanding and ability to perform. It is quite possible that a combination of these causal mechanisms leads to better performance. Both of these explanations are embedded within the spreading activation theory, which suggests that retrieving information also activates related concepts in memory in a chain reaction event that leads to an elaborate network of information being retrieved [37]. Indeed, Anderson [38] showed that generating elaborative structure for a term allowed for more retrieval cues and thus easier and more accurate retrieval of the information. Our results seem to lend support to this theory. Certainly further study may be able to further define this mechanism. Ultimately, if our goal is to accurately assess what students understand, and not just what they can recall, it appears that including additional items gives students an advantage and increases their ability to demonstrate their understanding.

A common final exam, consisting of both low-level and high-level items was given to both sections to determine if differences in overall learning occurred. Results showed that students given additional questions on unit exams led to higher scores on items assessing understanding of the material covered in the curriculum. Thus, not only do additional items allow more accurate assessment of student understanding and greater opportunity for students to successfully demonstrate that understanding, but it also appears to lead to more successful concept construction overall, as evidenced by higher achievement on the end of course assessment. These results suggested that lengthier exams taken by students in the extended condition may have given them additional practice and allowed for better performance on the final exam, an evidence of the testing effect. Indeed, Chan, McDermott, and Roediger [39] showed that testing on a subset of items enhanced recall of additional related items on the final exam. It is also likely that the exams served as a learning experience, as supported by several previous studies [40] and that the learning experience was greater with more extensive exams. Regardless of the exact cause, it is evident that more extensive exams led to greater concept attainment.

### Cognitive Fatigue

Our results support the findings of Ackerman and Kanfer [3] that students do not experience cognitive fatigue due to the length of the exam. We extend the applicability of these results to content-based, context-specific examinations. To confirm this, we analyzed performance of students in the extended condition on the first 50 questions of the exam compared to their performance on the second 50 questions. If students were experiencing cognitive fatigue, and assuming students completed the test in numerical order, we would expect lower performance on the second half of the exam. However, students performed equally or better on the second half of the exam as compared to the first. This seems to confirm that no cognitive fatigue was experienced, further justifying the inclusion of additional items. However, student comments suggest that there is a delicate balance between including enough items to accurately assess their knowledge and discouraging students by causing subjective fatigue with a lengthy exam. Comments were gathered from students in the extended condition in the form of course evaluations:

Student 1: “The tests were ridiculously long. I would get to the last couple of pages and be so burnt out that I didn't really focus and do my best.”

Student 2: “I really enjoyed the course but the 100 questions tests…ruined it for me.”

One of the major drawbacks of additional questions is the additional time that it takes to complete the exams. Students in the extended condition, on average, took one and a half times longer to complete the exam then students in the standard condition. This is likely a cause for many of the negative comments and reported subjective fatigue. Certainly increasing the number of items increases the minimum amount of time required to complete the exam. However, there were students in the standard condition that spent more time taking the standard-length exam than students who took the extended exam. Does additional time-on-task increase the students' exam scores? In other words, by giving longer exams, are we obligating students to spend an unreasonable amount of time taking the test? Interestingly, we found no correlation between time spent on the exam and exam performance, suggesting that additional time does not improve exam scores. However, this is a simplistic correlative study and does not take into account differences in student personalities, learning styles, or test-taking strategies that might account for differences in time needed to complete an exam.

### Educational Implications

Given the results of this study, it appears that additional items actually benefit students and give a more accurate assessment of student knowledge. However, one must consider the purpose of an introductory level, non-majors biology course. It is obvious that extensive, difficult exams are discouraging to students and promote subjective fatigue. Students in a non-majors course are particularly susceptible to discouragement being unfamiliar and sometimes afraid of the subject matter. This introductory course is often the only exposure to biology that they will experience. Thus, one must balance the desire to accurately assess with the goal of building a love for and fascination of biology. We suggest several possible solutions. First, more frequent testing would allow for fewer learning outcomes to be included on each exam and therefore more items to be included without making the exams overly extensive. Another solution may be to decrease the number of learning outcomes actually assessed while encouraging students to study as if all learning outcomes will be assessed. Interspersing low-level items within the difficult items has been shown to increase student satisfaction with exams (unpublished data), as this student pointed out:

Student 3: “I think keeping the tests 100 questions long, but varying the question types would be much easier on the students. [100] of those application questions is just horrible, and I would love some T/F or matching.”

However, low-level questions do not necessarily benefit their learning, nor do they assess learning outcomes designed around higher levels of thinking. Unfortunately, low-level recall items seem to be an expectation among students, particularly in the sciences.

In addition, multiple-choice testing was chosen for this study to maintain objectivity and to collect quantitative data; however, it may not be the optimal testing format; other test formats may prove to be less discouraging and give students a greater opportunity to demonstrate their knowledge, as suggested by some studies [41]–[43].

This study supports two main assessment strategies. First, increasing the number of items assessing each learning outcome increases the accuracy of the assessment. Second, extensive exams, although beneficial to student grades, in some cases discourage students and lead to feelings of resentment toward the subject. We have offered several solutions to balance more accurate assessment with student satisfaction. We encourage further research on best practices that can lead to accurate assessment while maintaining student satisfaction.

## References

[1] Wiggins G, McTighe J (1998) Understanding by design. Alexandria, VA: Association for Supervision and Curriculum Development.

[2] Thorndike RM (2005) Measurement and evaluation in psychology and education, 7^th^ ed. Upper Saddle River, New Jersey: Pearson Education, Inc.

[3] Ackerman PL, Kanfer R (2009) Test length and cognitive fatigue: An empirical examination of effects on performance and test-taker reactions. J Exp Psychol: Appl 15: 163–181. 19586255 10.1037/a0015719

[4] Ackerman PL, Kanfer R (2006) Test length and cognitive fatigue. Final report to the College Board. Atlanta, GA: Author.

[5] Arai T (1912) Mental fatigue. Contributions to Education 54: 1–115).

[6] Lewin K (1935) A dynamic theory of personality: Selected papers. (DK Adams & KE Zener, Trans.). New York: McGraw-Hill.

[7] Martyn GW (1913) A study of mental fatigue. Br J Psychol 5: 427–446.

[8] Davis DR (1946) This disorganization of behaviour in fatigue. J Neurol Neurosurg Psychiatry 9: 23–29. 20993389 10.1136/jnnp.9.1.23 PMC497917

[9] FairTest (2006) Scores from the “New” SAT expected to dip. FairTest: The National Center for Fair and Open Testing. Available: www.fairtest.org. Accessed: 18 Jul 2012.

[10] FOXNews (2006) Fatigue may be reason for dip in SAT scores. Associated Press story. Available: www.foxnews.com. Accessed 18 Jul 2012.

[11] Laitusis CC, Morgan DL, Bridgeman B, Zanna J, Stone E (2007) Examination of fatigue effects from extended-time accommodations on the SAT Reasoning Test. College Board Research Report No. 2007-1. New York: The College Board.

[12] Liu J, Allspach JR, Feigenbaum M, Oh H-J, Burton N (2004) A study of fatigue effects from the new SAT. College Board Research Report No. 2004-5. New York: The College Board.

[13] Karpicke JD, Roediger HL III (2008) The critical importance of retrieval for learning. Science 319: 966–968. 18276894 10.1126/science.1152408

[14] Carpenter SK, DeLosh EL (2006) Impoverished cue support enhances subsequent retention: Support for the elaborative retrieval explanation of the testing effect. Mem Cogn 34: 268–276. 10.3758/bf03193405 16752591

[15] Carpenter SK, Pashler H (2007) Testing beyond words: Using tests to enhance visuospatial map learning. Psychon Bull Rev 14: 474–478. 17874591 10.3758/bf03194092

[16] Carpenter SK, Pashler H, Cepeda NJ (2009) Using tests to enhance 8^th^-grade students' retention of U.S. history facts. Appl Cogn Psychol 23: 760–771.

[17] Carpenter SK, Pashler H, Wixted JT, Vul E (2008) The effects of tests on learning and forgetting. Mem Cogn 26: 438–448. 10.3758/mc.36.2.438 18426072

[18] Carrier ML, Pashler H (1992) The influence of retrieval on retention. Mem Cogn 20: 633–642. 10.3758/bf03202713 1435266

[19] Chan JCK, McDermott KB (2007) The testing effect in recognition memory: A dual process account. J Exp Psychol Learn Mem Cogn 33: 431–437. 17352622 10.1037/0278-7393.33.2.431

[20] Johnson CI, Mayer RE (2009) A testing effect with multimedia learning. J Educ Psychol 101: 621–629.

[21] McDaniel MA, Anderson JL, Derbish MH, Morrisette N (2007) Testing the testing effect in the classroom. Eur J Cogn Psychol 19: 494–513.

[22] Rohrer D, Taylor K, Sholar B (2010) Tests enhance the transfer of learning. J Exp Psychol Learn Mem Cogn 36: 233–239. 20053059 10.1037/a0017678

[23] Bybee R (1993) An instructional model for science education. Developing biological literacy. Colorado Springs, CO: Biological Sciences Curriculum Studies.

[24] Lawson AE (2002) Science teaching and the development of thinking. Belmont, CA: Wadsworth/Thomson Learning.

[25] Anderson LW, Krathwohl DR (2001) A Taxonomy for learning, teaching and assessing. New York: Longman.

[26] Bloom BS (1984) Taxonomy of educational objectives. Boston: Allyn and Bacon.

[27] Lawson AE (1978) The development and validation of a classroom test of formal reasoning. J Res Sci Teach 15: 11–24.

[28] Lawson AE, Alkhoury S, Benford R, Clark BR, Falconer KA (2000) What kinds of scientific concepts exist? Concept construction and intellectual development in college biology. J Res Sci Teach 37: 996–1018.

[29] Shi J, Wood WB, Martin JM, Guild NA, Vicens Q, et al (2010) A diagnostic assessment for introductory molecular and cell biology. CBE Life Sci Educ 9: 453–461. 21123692 10.1187/cbe.10-04-0055 PMC2995763

[30] Heiss ED, Obourn ES, Hoffman CW (1950) Modern science teaching. New York: Macmillan.

[31] Howard DR, Miskowski JA (2005) Using a module-based laboratory to incorporate inquiry into a large cell biology course. CBE Life Sci Educ 4: 249–260. 10.1187/cbe.04-09-0052 PMC1200779 16220145

[32] Jensen JL, Lawson AE (2011) Effects of collaborative group composition and inquiry instruction on reasoning gains and achievement in undergraduate biology. CBE Life Sci Educ 10: 64–73. 21364101 10.1187/cbe.10-07-0089 PMC3046889

[33] Minner DD, Levy AJ, Century J (2009) Inquiry-based science instruction: What is it and does it matter? Results from a research synthesis years 1984 to 2002. J Res Sci Teach 47: 474–496.

[34] Renner JW, Stafford DG, Coffia WJ, Kellogg DH, Weber MC (1973) An evaluation of the Science Curriculum Improvement Study. School Sci Math 73: 291–318.

[35] Rissing SW, Cogan JG (2009) Can an inquiry approach improve college student learning in a teaching laboratory? CBE Life Sci Educ 8: 55–61. 19255136 10.1187/cbe.08-05-0023 PMC2649651

[36] Spiro MD, Knisely KI (2008) Alternation of generations and experimental design: A guided-inquiry lab exploring the nature of the her1 developmental mutant of Ceratopteris richardii (C-Fern). CBE Life Sci Educ 7: 82–88. 18316811 10.1187/cbe.07-82-88 PMC2262118

[37] Collins AM, Quillian MR (1972) Experiments on semantic memory and language comprehension. In L Gregg (Ed.), Cognition and learning (pp. 117–138). New York, NY: Wiley.

[38] Anderson JR (1976) Language, memory, and thought. Hillsdale, NJ: Erlbaum.

[39] Chan JCK, McDermott KB, Roediger HL III (2006) Retrieval-induced facilitation: Initially nontested material can benefit form prior testing of related material. J Exp Psychol Gen 135: 553–571. 17087573 10.1037/0096-3445.135.4.553

[40] National Research Council (1996). National science education standards. Washington, DC: National Academy Press.

[41] Anbar M (1991) Comparing assessments of students' knowledge by computerized open-ended and multiple-choice tests. Acad Med 66: 420–422. 2059271 10.1097/00001888-199107000-00012

[42] Heyborne WH, Clarke JA, Perrett JJ (2011) A comparison of two forms of assessment in an introductory biology laboratory course. J Coll Sc Teach 5: 28–31.

[43] Traub RE, Fisher CW (1977) On the equivalence of constructed-response and multiple-choice tests. Appl Psychol Meas 1: 355–369.

